# Supplementary material for: A Comprehensive Assessment of Ultraviolet-Radiation-Induced Mutations in Flammulina filiformis Using Whole-Genome Resequencing
Source: J Fungi (Basel). 2024 Mar 20;10(3):228. doi: 10.3390/jof10030228 (PMC10971301; doi:10.3390/jof10030228)
Supplement: Supplementary file 1 [file jof-10-00228-s001.zip › Supplementary Material S8/KEGG annotation/out/64381550635650.os/KO/out_map/map05032.html]

KEGG PATHWAY: Morphine addiction - Reference pathway


|  |  |
| --- | --- |
| **Morphine addiction - Reference pathway** |  |

[
Pathway menu
| Organism menu
| Pathway entry
| Show description
| User data mapping
]

Morphine is an alkaloid from the plant extracts of opium poppy. Although morphine is highly effective for the treatment of pain, it is also known to be intensely addictive. We now know that the most important brain-reward circuit involves dopamine (DA) -containing neurons in the ventral tegmental area (VTA) of the midbrain and their target areas in the limbic forebrain, in particular, the nucleus accumbens (NAc) and frontal regions of cerebral cortex. Morphine can cause indirect excitation of VTA dopamine neurons by reducing inhibitory synaptic transmission mediated by GABAergic neurons. The chronic use of morphine is characterized by adaptive changes in neurons and neuronal communication; such adaptations (e.g., 'superactivation' of adenylyl cyclase) must underlie altered behaviour associated with morphine dependence and withdrawal syndrome, as well as drug-induced craving and relapse to drug use.

|  |  |
| --- | --- |
| Reference pathway | 100% |
